# P05.65. BHIP - be healthy in pregnancy: strategies nutritional and physical activity interventions to improve gestational weight gain management

**DOI:** 10.1186/1472-6882-12-S1-P425

**Published:** 2012-06-12

**Authors:** R Walji, S Atkinson, O Wahoush

**Affiliations:** 1 McMaster University, Hamilton, Ontario, Canada

## Purpose

Excess gestational weight gain (GWG) in pregnancy is a major clinical challenge affecting 55-75% of Canadian women who enter pregnancy overweight and about 40% women of normal weight. The adverse sequelae of excess GWG for both mother and child are well documented and include a number of adverse health outcomes (such as gestational diabetes, hypertension and preeclampsia), which impose substantive burden of our health system. The larger research program aims to conduct a randomized trial with a diet and exercise intervention for optimizing GWG. The project presented here is a preliminary phase in order to determine feasibility and patient preferences to the proposed intervention.

## Methods

This project uses a qualitative approach employing focus groups and interviews of participant women (pregnant or recently pregnant) and health care providers that aims to identify the preferred evidence-based strategies for women to effectively manage their GWG during and after pregnancy and how best to implement the selected intervention. Primary research question: What are the preferences of pregnant and post-partum women and their health providers for engaging in healthy eating and increased physical activity? Secondary questions include: What do pregnant or recently pregnant women and health providers identify as enablers or barriers that support or limit successful management of GWG? What are women’s and health providers’ perceptions of GWG in relation to their health and the health of the child? What approaches have women and health providers tried to manage excess GWG?

## Results

Outcomes include an identified preferred diet and exercise intervention for the planned clinical trial and information, which enables refinement of a locally acceptable implementation plan for the intervention.

## Conclusion

Collectively information from women and service providers enabled a comprehensive understanding of barriers, enablers and opportunities for the successful implementation of an intervention for GWG management.

